# Structure–Property Relationships in PHB-Based Copolymers and PHB/PLA Biocomposites Modified with Hydroxyapatite and Chitosan

**DOI:** 10.3390/polym18080913

**Published:** 2026-04-09

**Authors:** Yang Liu, Handuo Niu, Dongwei Li, Wei Nie, Ihor Semeniuk, Nataliia Koretska

**Affiliations:** 1 Laboratory of Polymer Materials for Extreme Conditions, Changchun Institute of Applied Chemistry, Chinese Academy of Sciences, Changchun 130022, China; 2 School of Chemistry and Environmental Engineering, Changchun University of Science and Technology, Changchun 130022, China; 3 Department of Physical Chemistry of Fossil Fuels of the Institute of Physical-Organic Chemistry and Coal Chemistry Named After L. M. Lytvynenko, National Academy of Sciences of Ukraine, 3a, Naukova Str., 79060 Lviv, Ukraine

**Keywords:** polyhydroxyalkanoates, polyhydroxybutyrate, nanohydroxyapatite, chitosan, tissue engineering

## Abstract

The challenge of substituting bone defects necessitates the search for effective biomaterials based on biopolymer composites with biocompatible fillers. A promising approach in bone tissue engineering is the use of regenerative scaffolds based on polyhydroxyalkanoates (PHAs), specifically poly(3-hydroxybutyrate)—P(3HB), which are characterized by high biocompatibility and osteoinductive potential. In this study, we evaluate the changes in the mechanical, thermal, and morphological properties of P(3HB) within P(3HB)-copolymers/HA, P(3HB)/CS, P(3HB)/PLA/CS, and P(3HB)/PLA/HA composites. These materials, containing various filler contents (up to 70 wt.% of HA–hydroxyapatite or CS–chitosan), were obtained using melt extrusion compounding. It is shown that the modification of biopolymer matrices promotes a decrease in melting temperature, improvement of mechanical characteristics, and an increase in material elasticity. At high filler concentrations, nanoparticle agglomeration and a deterioration of physical-mechanical properties were observed. It was established that a content of 10–20 wt.% of nano-hydroxyapatite and chitosan is optimal, as these composites most closely match the mechanical properties of bone tissue. The results obtained indicate the high potential of the developed nanocomposites for the creation of biodegradable implants in reconstructive orthopedics.

## 1. Introduction

Tissue engineering is one of the leading fields of biotechnology, which has been rapidly evolving for more than two decades [1,2]. Biodegradable and biocompatible polymers of natural or synthetic origin are considered some of the most promising biomaterials for fabricating scaffolds intended for bone tissue regeneration. A promising direction in this field is the development of hybrid materials based on biodegradable polymers. Although bone tissue possesses self-regenerative capabilities, critical defects require biocompatible, bioactive, and mechanically strong materials with a porous structure that promotes osseointegration without immune complications [3,4].

Understanding the physiological structure and composition of bone is crucial for the development of biomaterials, implants, and devices. Bone is a porous material consisting of organic and inorganic phases, as well as water. The inorganic phase is primarily hydroxyapatite, while the organic phase consists mainly of type I collagen. On average, bone contains about 70% mineral matter, 25% organic matter, and 5% water. This combination confers strength to the bone while simultaneously maintaining flexibility. Bones are classified into cortical and trabecular types, which share a similar composition but differ in porosity and strength. Therefore, bone properties depend not only on composition but also on internal structure. Moreover, the mechanical properties of human bone vary depending on bone type, ethnicity, sex, and age. Variations in mechanical properties are also observed within the same individual. Consequently, scaffolds must be tailored to the specific mechanical requirements of each application [5].

Implementing advanced technologies is an effective strategy for developing new materials for bone repair. Nanotechnology and 3D printing allow for the precise modification of material characteristics. Polyhydroxyalkanoates (PHAs), due to their thermoplasticity and favorable properties, represent a promising matrix for bioactive composites [6,7,8]. The most common representative of PHAs is poly(3-hydroxybutyrate) (P(3HB)), which is suitable for bone tissue engineering due to its biocompatibility and ability to maintain mechanical properties over time. PHB supports osseointegration without chronic inflammation and can withstand loads until replaced by native tissue. The thermoplasticity of P(3HB) makes it suitable for creating composites with bioactive fillers. However, its high crystallinity, low elasticity, and brittleness limit its application under dynamic loads [9]. Therefore, without modification, P(3HB) cannot provide the necessary flexibility or strength for most orthopedic and cardiovascular applications. Furthermore, poor processability and a narrow processing window can lead to thermal degradation during melt extrusion.

These limitations can be addressed by reducing the melting temperature, thereby lowering the required processing temperature and expanding the processing window. Blending with polymers that have lower melting points is a promising solution. Polymer blends are mixtures of at least two polymers or copolymers expected to exhibit synergistic advantages absent in the individual polymers. Blending techniques can significantly improve physical and mechanical properties. P(3HB) processability during filament extrusion can be enhanced by blending with polylactide (PLA), a biodegradable polymer with a lower melting point and better melt strength, or by preparing composites with PHB copolymers, such as P(3HB-co-4HB), P(3HB-co-3HV), or P(3HB-co-3HHx). These are biodegradable polyesters of microbial origin, characterized by specific thermal, mechanical, and rheological properties [10,11,12].

The thermal, physical, and mechanical characteristics of these polymers are essential for assessing material stability and processability. Differential Scanning Calorimetry (DSC) enables the analysis of thermal transitions, including melting, crystallization, glass transition, and heat capacity. The characteristics obtained via DSC correlate with the final material properties, including stiffness, strength, elongation at break, crystalline stability, elasticity, and processability. The effects of additives, fillers, polymer blends, and copolymerization are also studied using DSC [13]. Inorganic materials, such as tricalcium phosphate or hydroxyapatite, can be used as polymer fillers. Both minerals have a chemical composition similar to mammalian bone, with high calcium and phosphorus content, providing biocompatibility and osteoconductivity. Surface morphology and hydrophilic properties can be tuned by adjusting the tricalcium phosphate content, making these composites promising materials for bone regeneration [14]. The addition of chitosan increases the elasticity and reduces the brittleness of the material, while the incorporation of hydroxyapatite enhances the strength and stiffness of the composites, thereby improving their effectiveness in biomedical applications, particularly in bone tissue engineering [15,16].

Thus, the aim of this study is to evaluate structure–property relationships in PHB-based copolymers and PHB/PLA biocomposites modified with hydroxyapatite and chitosan using melt extrusion compounding.

## 2. Materials and Methods

### 2.1. Materials

The work used poly-3-hydroxybutyrate (P(3HB)), Mw = 480,000, melting temperature = 151 °C, density = 1.23 g/cm^3^, 98%, powder), CAS:26063-00-3 (Donggvuan Hongcheng Plactic Raw Materials Co., Ltd., Dongguan, Guangdong, China); poly-3-hydroxybutyrate-4-hydroxybutyrate (P(3HB-co-4HB)), 4HB content = 10 mol.%, melting temperature = 120 °C, powder, CAS:125495-90-1 (Donggvuan Hongcheng Plactic Raw Materials Co., Ltd., Dongguan, Guangdong, China); poly-3-hydroxybutyrate-3-hydroxyvalerate (P(3HB-co-3HV)), melting temperature = 155 °C, density = 1,25 g/cm^3^, 4HV contain = 10 mol.%, powder, CAS: 80181-31-3 (Donggvuan Hongcheng Plactic Raw Materials Co., Ltd., Dongguan, Guangdong, China); poly-3-hydroxybutyrate-3-hydroxyhexanoate (P(3HB-co-3HHx)), melting temperature = 132 °C, 3HHx content = 10 mol.%, powder, CAS:147398-31-0 (Icoman, Zhongcheng Plactic Materials Co., Ltd., Guangzhou, Guangdong, China); polylactide (PLA), Mw = 60,000, density = 1.24 g/cm^3^, melting temperature = 175 °C, powder, CAS:26100-51-6 (Adamas-beta, Shanghai, China); chitosan (CS) CAS:9012-76-4, Mw = 100,000, degree of deacetylation ≥ 95% (Adamas-beta, Shanghai, China); hydroxyapatite (HA), Mw = 502, 20 nm, 97.5% CAS:1306-06-5 (Adamas-beta, Shanghai, China).

### 2.2. Methods

#### 2.2.1. Melt Extrusion Compounding

P(3HB), P(3HB-co-4HB), P(3HB-co-3HV), P(3HB-co-3HHx), PLA, HA, and CS powders were dried in a vacuum oven to a constant weight. Composite blends were prepared by introducing modifiers into the P3HB matrix using a Thermo Scientific™ Process 11 laboratory parallel twin-screw extruder. The temperature profile of the extruder was set as follows: the loading zone was 60 °C, the second zone was 150 °C, the third was 155 °C, the fourth was 160 °C, the fifth was 170 °C, the sixth and seventh were 170 °C each, the eighth was 165 °C, and the extruder head temperature was 150 °C. The screw rotation speed was 60 rpm. At the extruder outlet, the polymer composites were obtained in the form of filaments. To evaluate the properties of the composites, the results were compared with the characteristics of pure polymers obtained under similar extrusion conditions.

The extrusion temperature profile was set 10–30 °C above the melting temperature of P(3HB) (150 °C), in accordance with literature recommendations for the processing of polyhydroxybutyrate. Such a temperature range ensures sufficient melt flow while minimizing the risk of thermal degradation, given the narrow processing window characteristic of P(3HB) [6,17].

#### 2.2.2. Scanning Electron Microscopy (SEM)

The microstructures of the filaments obtained from the biopolymers and their composites were studied using scanning electron microscopy. SEM allows for a detailed evaluation of the material’s structure at the micro- and submicron scales [18,19]. Filament samples (10.0 × 2.5 ± 0.15 mm) were fractured after cooling in liquid nitrogen. To prevent sample charging, gold sputtering was applied prior to analysis. The investigations were conducted using XL30 (Philips, Amsterdam, The Netherlands), Hitachi S-3000N (Tokyo, Japan), and Phenom Pure (Thermo Fisher Scientific, Eindhoven, The Netherlands) scanning electron microscopes. Part of the research was performed in one laboratory, while the subsequent analysis of the samples was carried out in another laboratory using alternative equipment due to the organizational and technical conditions of the experiment.

#### 2.2.3. Thermal Analysis (Thermogravimetric (TG) and Derivative Thermogravimetric (DTG) Analysis)

To assess the thermal stability of the obtained filaments, thermal analysis was performed on a Mettler Toledo TGA-1100 derivatograph (Greifensee, Switzerland) in the temperature range of 25–800 °C under a nitrogen atmosphere with a flow rate of 10 mL/min. The heating rate was 10 °C/min, and the average mass of the samples was 5 mg. The melting, crystallization, and glass transition temperatures of the samples were determined by differential scanning calorimetry (DSC) using a PerkinElmer DSC 4000 device (Shelton, CT, USA). The first heating scan was carried out in the temperature range from −50 to 200 °C at a rate of 10 °C/min under a nitrogen atmosphere with a flow rate of 10 mL/min, followed by an isothermal hold for 5 min. The first cooling scan was carried out from 200 to −50 °C at a rate of 10 °C/min. The samples were then kept for 5 min at a constant temperature. The second heating scan was carried out under a nitrogen atmosphere from −50 to 200 °C at a rate of 10 °C/min. The average mass of the DSC samples was 10 mg [20]. Differential scanning calorimetry (from –50 to 200 °C) was conducted in a temperature interval that encompasses all major thermal transitions of P(3HB), including glass transition, crystallization, and melting, while avoiding significant thermal degradation. A heating-cooling-second heating cycle was used to eliminate thermal history effects and to determine the intrinsic thermodynamic properties of the materials [21].

The temperature range for thermogravimetric analysis (25–800 °C) was selected to fully characterize the thermal decomposition behavior of the P(3HB)-based filaments and to determine the residual inorganic fraction (hydroxyapatite) following the complete degradation of the polymer matrix. Since the thermal decomposition of P(3HB) typically occurs in the range of 250–320 °C, the upper limit of 800 °C ensures an accurate assessment of the mineral residue and overall thermal stability. A heating rate of 10 °C/min was applied to obtain reproducible degradation profiles.

#### 2.2.4. Mechanical Properties

The mechanical properties of the polymer filaments under tension were determined according to the ASTM D638 standard [22] using a Kimura Machinery RT-601U universal testing machine at a sample extension speed of 50 mm/min. Their tensile strength and relative elongation at break were determined. Shore D hardness measurements were carried out according to the ASTM D2240 [23] standard using a digital Shore D durometer at a load of 5 kg.

#### 2.2.5. FTIR Analysis

To confirm the formation of intermolecular hydrogen bonds between the components of the studied systems, FTIR analysis was performed using a Bruker INVENIO-R Fourier spectrometer (Ettlingen, Germany). The spectra were recorded in the wavenumber range of 4000–400 cm^−1^ with a resolution of 4 cm^−1^ at 128 scans. The measurements were performed using a diamond ATR crystal.

#### 2.2.6. Statistical Analysis

Each sample was measured five times. The results are presented as arithmetic means ± standard deviations. Statistical data processing was performed using standard Microsoft Excel methods.

## 3. Results and Discussion

### 3.1. Filament Production

**Figure 1:**
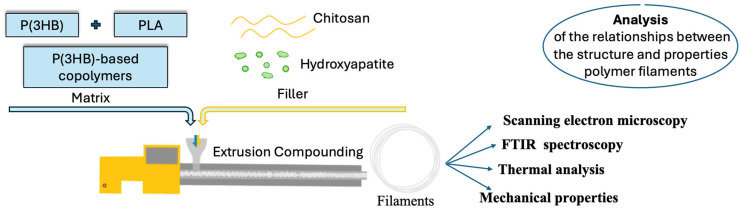
The experimental design of the study.

Filaments of polymer composites (P(3HB)-based copolymers and P(3HB)/PLA blends with 10–70 wt.% hydroxyapatite (HA) or chitosan (CS)) were fabricated via melt extrusion compounding. Figure 1 presents the experimental design of the study, which allowed us to evaluate the relationship between fracture morphology and the mechanical and thermal properties of the resulting composites.

### 3.2. Scanning Electron Microscopy of the Obtained Polymer Composite Filaments

SEM micrographs allowed us to characterize the fracture surface morphology of the filaments, the microstructure of the materials, and their failure mechanisms.

#### 3.2.1. SEM Images of P(3HB), PLA, and P(3HB)/PLA (50/50 wt.%)

**Figure 2:**
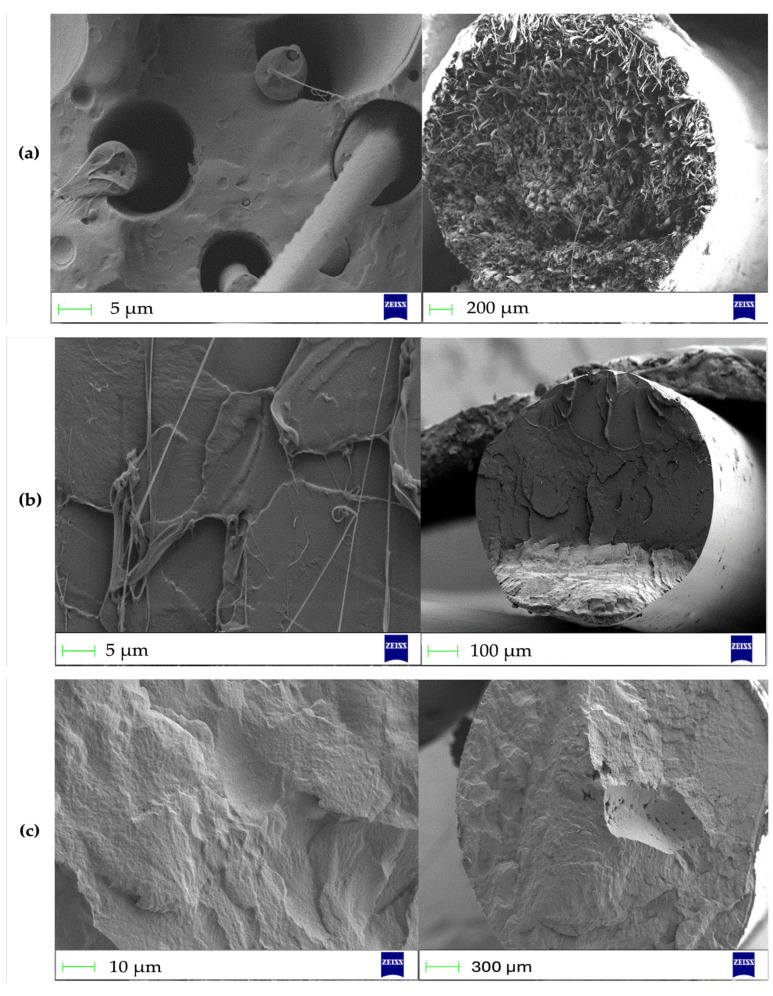
SEM images of the fracture surfaces of the filaments: (**a**) P(3HB), (**b**) PLA, and (**c**) P(3HB)/PLA (50/50 wt.%).

The fracture surfaces of the filaments made of P(3HB), PLA, and P(3HB)/PLA (50/50) are shown in Figure 2.

Polyhydroxybutyrate (P(3HB)) is characterized by a well-developed, porous, and fibrillar morphology of the cryogenic fracture surface (Figure 2a). Such a structure indicates the implementation of microplastic deformation mechanisms, specifically the drawing and rupture of fibrils, which results from the high crystallinity of the polymer. In contrast, polylactide (PLA) demonstrates a brittle fracture pattern typical of amorphous polymers, manifested as a predominantly smooth fracture surface with clearly defined crack propagation zones (Figure 2b).

The PHB/PLA (50/50) blend exhibits a two-phase morphology characteristic of partially compatible systems, combining features of both components. The heterogeneity of the fracture surface and the presence of voids after the detachment of dispersed domains indicate phase separation and insufficient interfacial adhesion (Figure 2c). Fracture occurs via a combined mechanism: cracks are initiated at the phase boundary and propagate with subsequent domain debonding, accompanied by local plastic deformation of the matrix. This fracture behavior is consistent with the limited compatibility of PHB and PLA in the absence of compatibilizers.

#### 3.2.2. SEM Images of P(3HB)/PLA/CS (45/45/10 and 35/35/30 wt.%)

**Figure 3:**
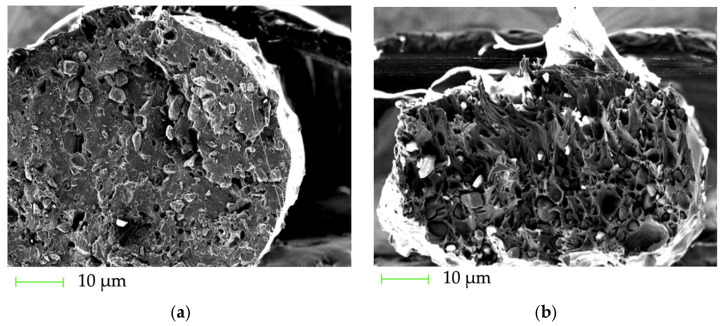
SEM micrographs of the fracture surfaces of P(3HB)/PLA/CS filaments: (**a**) 45/45/10 wt.% and (**b**) 35/35/30 wt.%; the scale 200 µm.

The fracture surfaces of the P(3HB)/PLA/CS filaments with filler contents of 10 wt.% and 30 wt.% are shown in Figure 3.

A significant influence of CS on the morphology and fracture mechanisms was observed. At a filler content of 10 wt.%, a rougher and more structured surface is formed (Figure 3a), which correlates with a slight decrease in tensile strength from 22.3 MPa for P(3HB) to 21.1 MPa, and in elongation from 3.8% to 3.6%. This indicates a partial suppression of brittle fracture. At a filler content of 30 wt.%, the appearance of cavities and agglomerates (Figure 3b) is accompanied by a sharp deterioration in mechanical properties: the strength decreases to 19.7 MPa, and the elongation to 2.0%. This indicates an increase in internal stresses and a brittle fracture mechanism. Thus, a moderate filler content contributes to a more energy-intensive fracture mechanism, while an excess leads to the structural heterogeneity of the composite.

#### 3.2.3. SEM Images of P(3HB)/PLA/HA (45/45/10, 40/40/20, and 35/35/30 wt.%)

**Figure 4:**
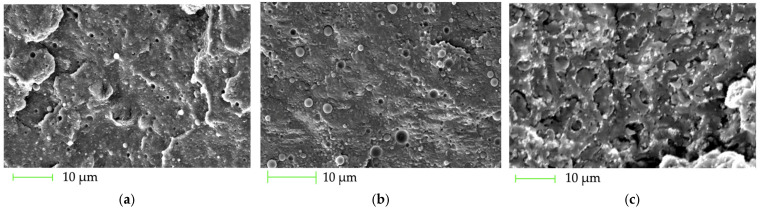
SEM images of the fracture surfaces of the P(3HB)/PLA/HA filaments: (**a**) 45/45/10 wt.%, (**b**) 40/40/20 wt.%, and (**c**) 35/35/30 wt.%; the scale 10 µm.

SEM analysis of the fracture surfaces of the P(3HB)/PLA/HA filaments (Figure 4) revealed a systematic change in morphology with increasing hydroxyapatite (HA) content and its direct correlation with mechanical properties.

The P(3HB)/PLA (50/50) matrix is characterized by a relatively smooth fracture surface with local deformation zones, corresponding to a high strength of 42.5 MPa and an elongation of 6.8%.

In the composition with 10 wt.% HA, the appearance of distinct microinclusions and characteristic particle pull-out zones is observed; however, the particles are generally uniformly distributed, and the number of pores and defects remains moderate (Figure 4a). Thus, a moderately rough surface is formed with a disperse distribution of particles and limited microcracking. Such morphology corresponds to only a slight decrease in mechanical properties (40.0 MPa; 5.6%).

At 20 wt.% HA, the key features characteristic of the 10 wt.% sample are preserved—in particular, the presence of local particle pull-out and a rough topography. At the same time, SEM demonstrates a qualitative change in the intensity of these effects: the number of particle pull-out zones increases, more developed cavities appear following the detachment of agglomerated particles, and signs of local matrix debonding are observed (Figure 4b). The increase in the scale of defects corresponds to a more noticeable decrease in ductility (3.9%) and strength (37.8 MPa).

A further increase in the HA content to 30 wt.% leads to massive particle agglomeration and the formation of significant porosity, which causes a predominantly brittle fracture behavior (Figure 4c). This is accompanied by a substantial decrease in strength (31.3 MPa) and elongation at break (2.8%), corresponding to failure controlled by filler defects.

#### 3.2.4. SEM Images of P(3HB)/HA (80/20, 50/50, and 30/70 wt.%)

**Figure 5:**
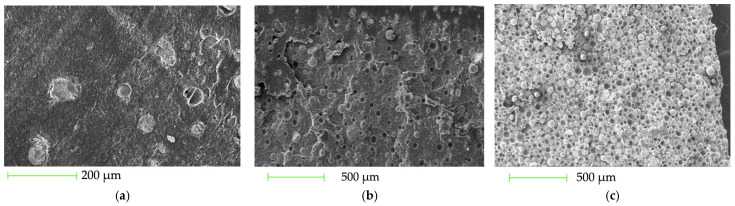
SEM images of the fracture surfaces of P(3HB)/HA filaments: (**a**) 80/20 wt.%, (**b**) 50/50 wt.%, and (**c**) 30/70 wt.%.

SEM analysis of the fracture surfaces of P(3HB)/HA composites (Figure 5) demonstrated a clear correlation between morphology and mechanical properties, depending on the hydroxyapatite content.

For unfilled P(3HB), which is characterized by a predominantly brittle fracture, the tensile strength is 34.2 MPa with a relative elongation of 3.8%. With the introduction of 20 wt.% HA, the formation of a relatively homogeneous, moderately rough surface with dispersed filler particles (Figure 5a) corresponds to a slight decrease in strength to 32.4 MPa and an elongation of 2.5%. This indicates the preservation of a mixed fracture mechanism with effective energy dissipation. At 50 wt.% HA, the development of porosity, interfacial defects, and traces of particle pull-out (Figure 5b) correlates with a further decrease in strength to 30.5 MPa and elongation to 2.4%, due to stress concentration in defect zones. A further increase in HA content up to 70 wt.% leads to filler agglomeration and the dominance of a brittle fracture mechanism (Figure 5c). This is accompanied by a sharp drop in strength to 21.7 MPa and a minimum elongation of 2.3%, confirming the loss of the composite’s capacity for plastic deformation.

#### 3.2.5. SEM Images of P(3HB-co-3HV)/HA (10 mol.% 3HV) (80/20, 50/50, and 30/70 wt.%)

**Figure 6:**
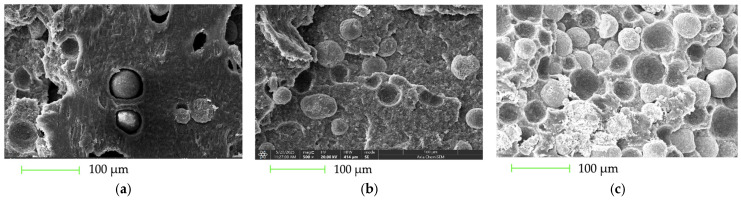
SEM images of the fracture surfaces of P(3HB-co-3HV)/HA filaments: (**a**) 80/20 wt.%, (**b**) 50/50 wt.%, and (**c**) 30/70 wt.%. The copolymer contains 10 mol.% 3HV; the scale 100 µm.

SEM analysis of the fracture surfaces of filaments based on the P(3HB-co-3HV) copolymer (containing 10 mol.% 3HV units) filled with hydroxyapatite revealed significant microstructural changes as the concentration of the inorganic phase increased from 20 to 70 wt.% (Figure 6). These changes directly correlate with the mechanical characteristics of the materials.

For the unfilled P(3HB-co-3HV) copolymer, a relatively homogeneous fracture is observed, corresponding to a tensile strength of 28.8 MPa and an elongation at break of 5.5%, which is typical for semi-crystalline PHA materials with moderate plasticity. With the introduction of 20 wt.% hydroxyapatite, a dense polymer matrix with evenly distributed spherical filler particles and occasional pores is observed (Figure 6a). This suggests sufficient interfacial adhesion and a mixed fracture mechanism involving plastic deformation of the polymer. This morphology is consistent with a slight decrease in strength to 26.9 MPa while maintaining a relatively high elongation (5.0%), indicating effective load transfer between phases.

At an HA content of 50 wt.%, the structure becomes more porous and heterogeneous, with numerous traces of particle pull-out and local interfacial defects (Figure 6b). The loss of continuity in the polymer phase causes a decrease in strength to 23.5 MPa and elongation to 4.2%, indicating an increased role of brittle fracture mechanisms and stress concentration in the filler zones. A further increase in HA content to 70 wt.% leads to particle agglomeration and the formation of a highly porous, granular structure with a minimal amount of polymer binder (Figure 6c). Failure occurs mainly along phase boundaries and defect zones, causing a sharp deterioration in mechanical properties: strength decreases to 19.1 MPa and elongation at break to 3.5%, typical of brittle composite systems.

Thus, SEM studies confirm that an increase in hydroxyapatite content promotes a transition from a relatively energy-intensive mixed fracture mechanism to a dominant brittle failure due to porosity and filler agglomeration. The optimal combination of morphological uniformity and mechanical properties is observed at 20 wt.% HA, whereas excessive filler concentration leads to the degradation of the composites’ structural integrity.

### 3.3. Thermal Analysis of Polymers and Composites

When analyzing polymers, their thermal stability should be studied. This is an important parameter that reflects the maximum temperature that the polymer can withstand before thermal decomposition. The thermal properties of filament samples were studied using TGA and DTG analyses.

#### 3.3.1. Thermal Analysis of P(3HB) and P(3HB)/CS Composites

With an increase in chitosan content within the P(3HB) matrix, the melting temperature of P(3HB) decreased from 150.87 °C to 150.33 °C at 10% CS content, to 149.09 °C at 20% CS content, and to 147.83 °C at 30% CS content. The enthalpy of melting (ΔHm) also decreased accordingly in the sequence of 60.18, 58.79, 47.73, and 40.18 J/g. Consequently, the introduction of chitosan into P(3HB) in amounts up to 30 wt.% leads to a change in the degree of crystallinity of the polymer matrix. At a filler content of 10 wt.%, CS causes an increase in the crystallinity of P(3HB). Meanwhile, at CS contents of 20–30 wt.%, the crystallinity of the polymer phase decreases, evidently due to the restriction of polymer chain mobility and the disruption of crystallization processes.

#### 3.3.2. Thermal Analysis of the P(3HB)/PLA Blend and P(3HB)/PLA/CS Composite

When mixing P(3HB) with PLA in a 50/50 ratio, the melting temperature (Tm) of P(3HB) decreased by 3.5 °C—from 151.2 to 147.7 °C. This indicates a disruption of its crystalline structure due to intermolecular interactions with PLA and the formation of less perfect crystallites (Table 1). Such a shift is a characteristic sign of partial polymer compatibility and correlates with the improvement of the composition’s mechanical properties. The melting enthalpy (ΔHm) of P(3HB) in the blend also decreased substantially—from 57.6 to 26.9 J/g—pointing to a significant reduction in the degree of crystallinity of P(3HB) caused by the inhibition of crystallization processes under the influence of PLA. The growth of the amorphous phase fraction enhances the material’s deformability and the overall plasticity of the composite.

The addition of chitosan (CS) to the P(3HB)/PLA matrix in amounts of 10 and 30 wt.% leads to a decrease in the melting temperature of the P(3HB) phase to 146.0 and 140.9 °C, respectively, as well as a reduction in melting enthalpy to 20.3 and 20.0 J/g. This decline in thermal and caloric parameters indicates a substantial suppression of P(3HB) crystallization in the presence of chitosan due to spatial restrictions on crystallite growth and intermolecular interactions between the polyester matrix and the functional groups of chitosan. Consequently, a more amorphous composite structure with a reduced degree of crystallinity is formed.

Conversely, at a chitosan content of 20 wt.%, the thermograms reveal the formation of a single distinct cold crystallization peak at 58.4 °C, a melting peak at 137.7 °C with a significant increase in melting enthalpy to 49.4 J/g, and the appearance of a single peak for the maximum rate of thermal degradation at 274.3 °C. This behavior may suggest that the P(3HB)/PLA system reaches a high degree of homogenization in the presence of chitosan, where phase separation is substantially reduced or disappears, and the components form a more coordinated crystalline structure. Likely, at this concentration, chitosan acts as an interfacial compatibilizer, improving the interaction between polymer phases and promoting the formation of joint or interpenetrating crystalline domains, resulting in enhanced thermal stability and an anomalously high melting enthalpy.

For pure P(3HB), the glass transition temperature (Tg) during the first heating cycle is 57.95 °C, while during the second heating, it drops sharply to 2.43 °C. This difference is associated with the relaxation of internal stresses and the rearrangement of the amorphous phase after the first thermal cycle. In the initial sample, the polymer exists in a non-equilibrium state following processing, leading to an inflated Tg. After melting and controlled cooling, a more mobile, thermodynamically stable amorphous phase is formed with a lower glass transition temperature, close to the characteristic value for P(3HB). For PLA, a less pronounced change in Tg is observed—from 64.59 to 60.72 °C—upon reheating, indicating partial structural relaxation without the radical rearrangement of the amorphous phase typical of more rigid polyesters with slower segmental mobility.

The P(3HB)/PLA (50/50) blend demonstrates behavior similar to P(3HB): Tg decreases from 63.88 °C to 2.65 °C after the first heating cycle. Such a sharp shift indicates the dominant influence of the P(3HB) phase on relaxation processes in the amorphous region of the blend and a significant rearrangement of interfacial morphology. This may also suggest partial compatibility of the system, where thermal treatment leads to the formation of a more mobile amorphous phase enriched with P(3HB) segments.

The obtained DSC results indicate a significant effect of chitosan on the relaxation properties of the amorphous phase in P(3HB)/PLA composites. The introduction of 10 wt.% chitosan leads to an increase in the glass transition temperature during the first heating to 66.8 °C compared to the unfilled matrix. This is due to the formation of intermolecular hydrogen bonds between the functional groups of chitosan and the ester groups of the polymers, thereby restricting the segmental mobility of the macrochains. As the filler content increases to 30 wt.%, Tg drops to 58.9 °C, suggesting partial agglomeration of chitosan and disorganization of the amorphous phase, accompanied by an increase in free volume and decreased efficiency of interfacial interaction. For the composition with 20 wt.% chitosan, a Tg value of 62.4 °C was obtained, corresponding to the optimal degree of system homogenization.

After reheating, a sharp decrease in the glass transition temperature to 2–4 °C is observed for all samples, which is related to thermal structural relaxation, the relief of internal stresses, and the formation of a more mobile amorphous phase controlled predominantly by the polyhydroxybutyrate component. Overall, chitosan acts as an effective structural modifier of the thermal behavior of P(3HB)/PLA composites, with the optimal balance of thermal stability and homogeneity achieved at moderate concentrations (approximately 20 wt.%).

#### 3.3.3. Thermal Analysis of P(3HB) and the P(3HB)/PLA/HA Composite

The introduction of hydroxyapatite (HA) into the P(3HB)/PLA matrix in amounts of 10, 20, and 30 wt.% resulted in a non-monotonic change in the melting temperature of P(3HB). At 10 wt.% HA content, the melting temperature increased from 147.7 °C to 149.69 °C, which may be attributed to the nucleating effect of the filler and the formation of more ordered crystalline structures. A further increase in HA content to 20 and 30 wt.% led to a decrease in the melting temperature to 143.55 and 141.10 °C, respectively, indicating a disruption of the polymer matrix crystallization due to the excessive presence of the inorganic phase. A similar trend was observed for the melting enthalpy: upon introducing 10 wt.% HA, its value increased from 26.88 to 31.17 J/g, confirming an increase in the degree of crystallinity of P(3HB), whereas further increases in filler content caused the enthalpy to decrease to 24.43 and 21.66 J/g, indicating the inhibition of crystalline phase formation.

#### 3.3.4. Thermal Analysis of P(3HB-co-4HB) and the P(3HB-co-4HB)/HA Composite

The introduction of hydroxyapatite into the P(3HB-co-4HB) matrix in amounts of 20, 50, and 70 wt.% resulted in a gradual decrease in the crystallization, melting, and thermal degradation temperatures of the composites. Specifically, the respective values changed from 60, 120, and 273 °C for the unfilled copolymer to 57, 120, and 270 °C at 20 wt.% HA; 55, 119, and 266 °C at 50 wt.% HA; and 54, 117, and 263 °C at 70 wt.% HA. The decrease in these temperatures with increasing filler content is due to the inhibition of crystallization processes and a reduction in the thermal stability of the copolymer matrix caused by spatial restrictions on crystallite growth, reduced macromolecular mobility, and the possible catalysis of polyester thermal degradation by the active surface of hydroxyapatite. At high loading levels, the polymer phase partially loses continuity, and the composite acquires characteristics of mineral-filled systems.

#### 3.3.5. Thermal Analysis of P(3HB-co-3HV) and the P(3HB-co-3HV)/HA Composite

Upon adding 20, 50, and 70% HA to the polyhydroxybutyrate-co-polyhydroxyvalerate P(3HB-co-3HV) matrix, the crystallization, melting, and degradation temperatures changed from 66, 155, and 268 °C to 70, 154, and 265 °C at 20% HA; 71, 151, and 263 °C at 50% HA; and 70, 148, and 259 °C at 70% HA. Changes in the crystallization, melting, and thermal degradation temperatures of P(3HB-co-3HV)/HA composites with increasing hydroxyapatite content (20–70 wt.%) are driven by the competition between the nucleating effect of the mineral filler and the spatial restriction of crystallite growth in the copolymer matrix. At moderate HA content, nucleation dominates, raising the crystallization temperature, whereas at high loading levels, the destabilizing effect on the polymer’s crystalline and thermal stability intensifies.

#### 3.3.6. Thermal Analysis of P(3HB-co-3HHx) and the P(3HB-co-3HHx)/HA Composite

Upon adding 20, 50, and 70% HA to the polyhydroxybutyrate-co-polyhydroxyhexanoate P(3HB-co-3HHx) matrix, the crystallization, melting, and degradation temperatures changed from 50, 132, and 263 °C to 53, 131, and 258 °C at 20% HA; 54, 129, and 256 °C at 50% HA; and 52, 127, and 252 °C at 70% HA. The introduction of hydroxyapatite into the P(3HB-co-3HHx) matrix leads to a complex effect: in the initial stages (low and moderate HA content), HA particles improve nucleation, which increases the crystallization temperature. However, at high HA content (50–70%), the restrictive effect dominates, disrupting the regularity of the crystalline structure and lowering both the melting temperature and the thermal stability of the polymer. Thus, the results indicate that the optimal HA content must be carefully balanced to achieve the desired thermal and mechanical properties of the composite.

### 3.4. Mechanical Properties of Polymers and Composites

#### Mechanical Properties of P(3HB), P(3HB)/PHA Copolymers, and P(3HB)/PHA/HA Composites

The mechanical properties of the polymers and composites were evaluated based on tensile strength, elongation at break, and Shore D hardness parameters.

**Figure 7:**
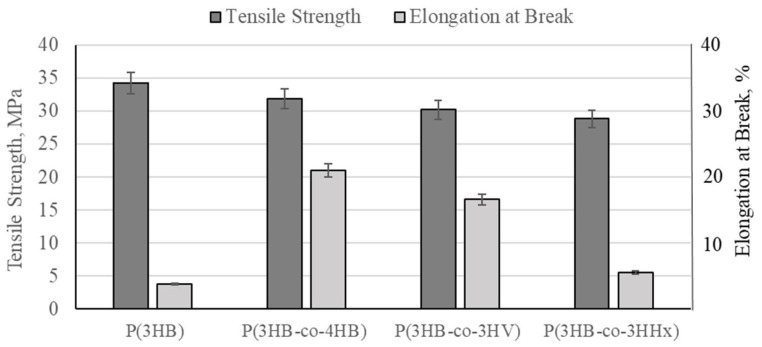
Mechanical properties of filaments of P(3HB)-based copolymers: the copolymers contain 90 mol.%. P(3HB).

For pure P(3HB) samples, the tensile strength was determined to be 34.2 MPa, which corresponds to a high level among biodegradable polymers, alongside a low elongation at break (3.8%), confirming the brittleness of the polymer (Figure 7).

Copolymerization of P(3HB) with more branched PHAs significantly increases plasticity: the introduction of 10% hydroxyvalerate (3HV), hydroxyhexanoate (3HHx), and 4-hydroxybutyrate (4HB) units increases the elongation to 5.5%, 16.6%, and 21%, respectively, while simultaneously decreasing the strength to 28.8, 30.2, and 31.9 MPa. The introduction of 10% 3HV units reduces the brittleness of P(3HB), but due to the preservation of high crystallinity, it does not provide sufficient elasticity. The inclusion of 10% HHx monomers lowers crystallinity and provides a significant increase in the elasticity of the copolymer without a substantial loss of strength. The addition of 10% 4HB units enhances the flexibility and elasticity of the P(3HB-co-4HB) copolymer, making it promising for biomedical applications.

The introduction of 70% hydroxyapatite (HA) into the P(3HB), P(3HB-co-4HB), P(3HB-co-3HV), and P(3HB-co-3HHx) copolymers decreases the tensile strength by a factor of 1.5–2.0 and the elongation at break by a factor of 1.4–1.8, indicating a reduction in the plasticity of the materials.

Introducing HA into the P(3HB) polymer matrix in amounts of 10, 20, and 30 wt.% leads to a gradual increase in Shore D hardness from 74.0 to 76.5, 78.5, and 79.5 hardness units, respectively (Table 2). This trend is driven by the high stiffness of the inorganic phase and its reinforcing effect, which restricts the mobility of polymer chains and increases the material’s resistance to local deformation. The increase in hardness indicates the formation of a more rigid and dense composite structure, as well as the universal effect of HA as a reinforcing additive that enhances surface hardness. However, in combination with SEM data and mechanical testing, this may point to a transition towards a more brittle fracture behavior at high filler concentrations.

The introduction of 30% chitosan into the P(3HB) matrix reduced the elongation at break from 3.8% to 2.0%. This is explained by the formation of hydrogen bonds that restrict the mobility of polymer chains, and at high concentrations, chitosan acts as a rigid filler. The slight decrease in tensile strength from 22.3 to 19.7 MPa is associated with partial incompatibility between the components.

**Figure 8:**
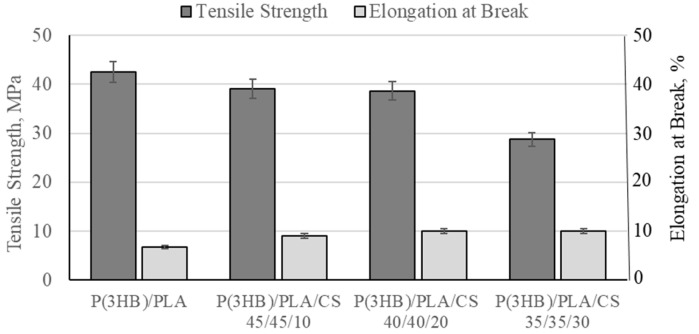
Effect of chitosan content on the mechanical properties of a composite based on poly-3-hydroxybutyrate and polylactic acid.

Conversely, in the more amorphous P(3HB)/PLA system, the addition of 30% chitosan increased the relative elongation from 6.8% to 10.0% and decreased the tensile strength from 42.5 to 28.7 MPa (Figure 8). This is due to a reduction in crystallinity and the weakening of interchain interactions, which leads to an increase in elasticity and the material’s deformability. Unlike P(3HB), the P(3HB)/PLA matrix has a lower degree of crystallinity due to the amorphous nature of PLA.

The obtained results indicate that the thermal behavior of the P(3HB)/PLA/chitosan system is determined by a combination of factors, including the degree of chitosan dispersion, the morphology of the composite, and the balance between interfacial interactions and aggregation processes.

At a low chitosan content (10 wt.%), the number of functional groups is insufficient to form an extensive network of intermolecular interactions, which limits their influence on the relaxation properties of the amorphous phase. Conversely, at a high content (30 wt.%), chitosan particle agglomeration processes dominate, leading to structural heterogeneity and a decrease in the efficiency of interfacial interaction.

At a content of 20 wt.%, an optimal balance is achieved between the degree of filler dispersion and the intensity of intermolecular interactions (particularly hydrogen bonds), which ensures a more uniform morphology and a maximum impact on the segmental mobility of the polymer chains. Thus, at a chitosan content of 20%, a balance is achieved between the structural organization and interfacial interaction of the P(3HB)/PLA/chitosan system.

The introduction of 30% hydroxyapatite into the P(3HB)/PLA matrix decreased the tensile strength from 42.5 to 31.3 MPa and the relative elongation at break from 6.8% to 2.8%, indicating a decrease in the plasticity of the polymer system. It is worth noting that chitosan reduced the tensile strength to a greater extent compared to hydroxyapatite, which is explained by the differences in their mechanical properties and interfacial interactions with the matrix. Chitosan contributes to a decrease in the crystallinity of P(3HB)/PLA, which negatively affects strength. Furthermore, it has limited compatibility with hydrophobic polyesters, leading to phase segregation and the formation of interfacial defects that facilitate crack initiation. Hydroxyapatite, on the contrary, often acts as a crystal nucleation center and can partially maintain or even improve structural integrity by interacting better with the polar groups of polyesters.

Thus, our findings demonstrate that the studied composites are promising materials for bone tissue engineering. While their expected biocompatibility is supported by established literature [24,25,26], future work will include cytotoxicity tests to experimentally verify this.

### 3.5. FTIR Analysis of Composites with Chitosan

To confirm the presence of hydrogen bonds in chitosan composites, FTIR spectra of the materials were taken.

**Figure 9:**
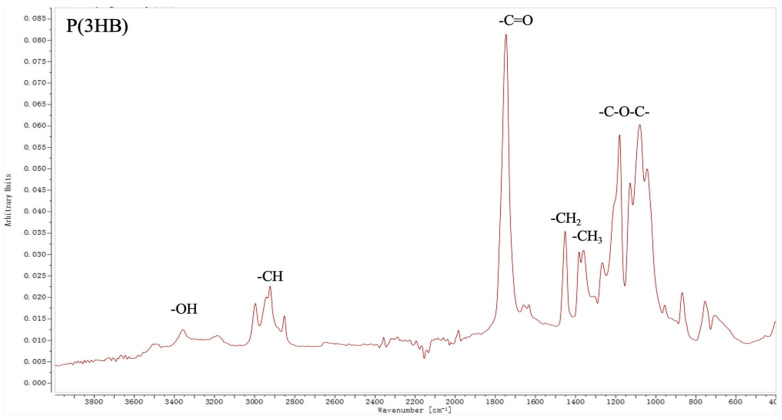

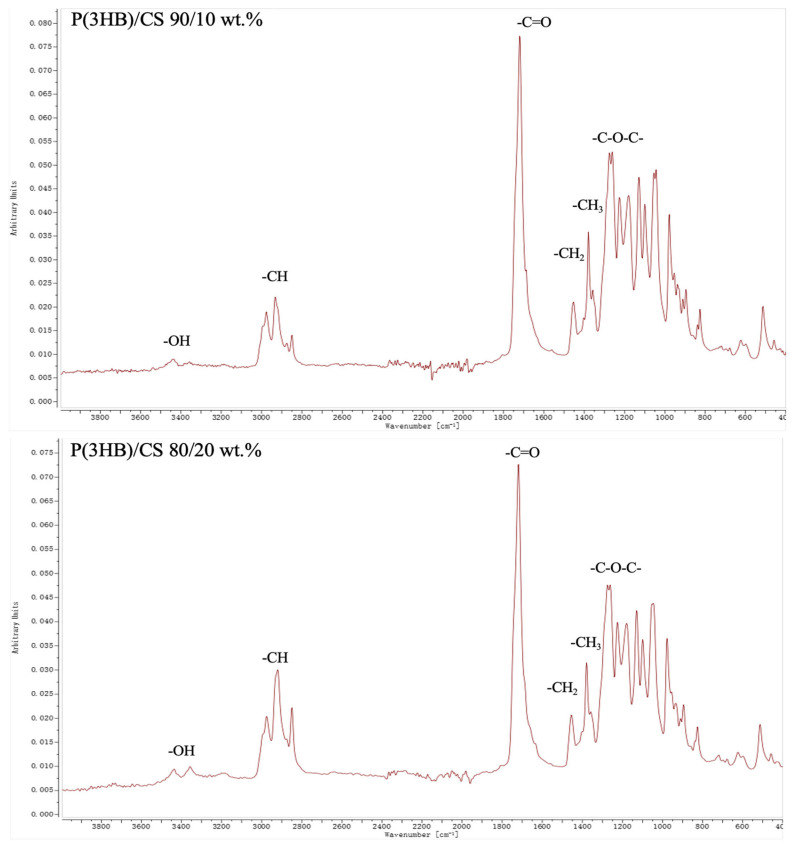
FTIR spectra of P(3HB) and its composites with chitosan.

The FTIR spectra of P(3HB) and P(3HB)/CS blends with varying compositions (10 and 20% CS) are shown in Figure 9. The decrease in the intensity of the absorption peak at 1750 cm^−1^, corresponding to the C=O carbonyl band, in the FTIR spectra of the P(3HB)/CS composites compared to pure P(3HB) can be justified by the formation of intermolecular hydrogen bonds between the system’s components. In pure P(3HB), the carbonyl groups are predominantly in a “free” state, resulting in a higher absorption band intensity. Upon the introduction of chitosan, which contains functional –OH and –NH_2_ groups, the formation of C=O···H–N and C=O···H–O type hydrogen bonds occurs. As a result, a portion of the P(3HB) carbonyl groups becomes involved in intermolecular interactions, which alters their dipole moment and reduces the efficiency of IR absorption. This is consistent with the formation of a more integrated polymer structure, where the P(3HB) carbonyl groups are partially “bound” via hydrogen bonds to the functional groups of chitosan. Thus, the decrease in the intensity of the carbonyl band can be considered as indirect confirmation of the formation of hydrogen bonds between P(3HB) and CS, and consequently, an improvement in their intermolecular interaction.

The decrease in band intensity in the 1050–1150 cm^−1^ region for the P(3HB)/CS composites compared to pure P(3HB) may be associated with the participation of ether (C–O–C, C–O) groups in intermolecular interactions with chitosan (Figure 9). The hydroxyl (–OH) and amine (–NH_2_) groups of chitosan are capable of forming hydrogen bonds not only with the carbonyl groups of P(3HB), but also with the oxygen atoms of the ether bonds, leading to a change in electron density and a decrease in the dipole moment of the corresponding vibrations. As a result, the band intensity decreases as the CS content rises, indicating an increase in the number of intermolecular contacts. Thus, the reduction in the intensity of the C–O–C and C–O bands confirms the involvement of these groups in hydrogen bonding and additionally points to the formation of interphase interactions between P(3HB) and chitosan.

The increase in band intensity in the 2880–3100 cm^−1^ range with rising CS content is driven by the increased contribution of the C–H and N–H vibrations of chitosan (Figure 9), as well as structural restructuring of the system due to the intermolecular interaction of the components.

**Figure 10:**
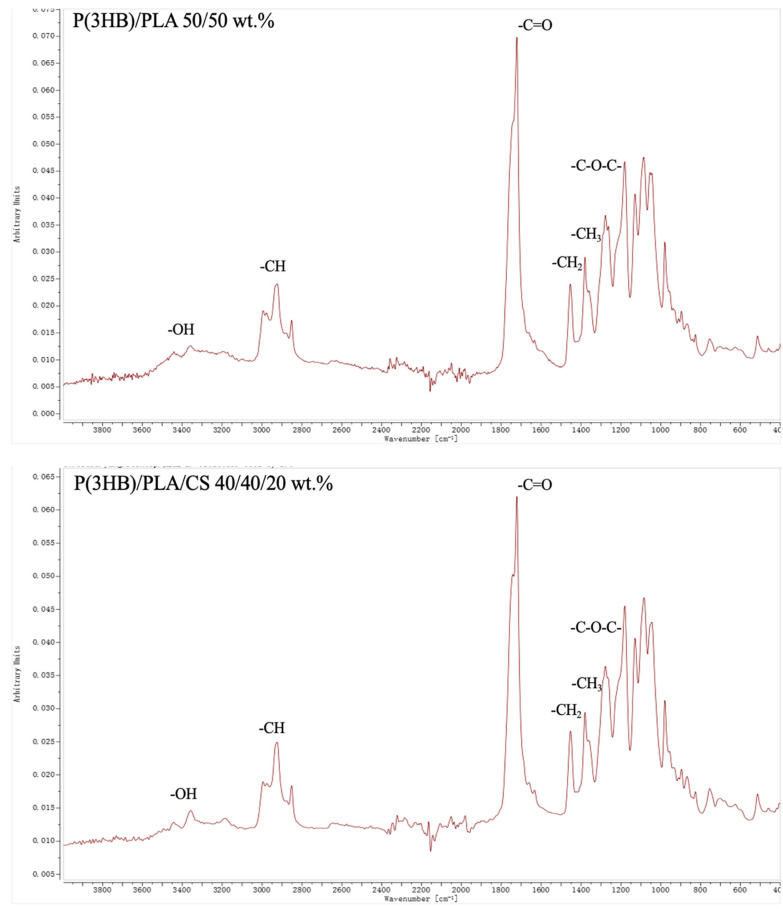
FTIR spectra of P(3HB)/PLA and P(3HB)/PLA/CS composites.

A similar trend is observed for P(3HB)/PLA systems and their composites with chitosan (10–30 wt.%) (Figure 10).

With an increase in CS content, a decrease in the intensity of bands at 1750 cm^−1^ and 1050–1150 cm^−1^ is noted, which is attributed to the increased contribution of the vibrations of the C–H and N–H groups of chitosan. In addition to the compositional effect, this may also be linked to the formation of hydrogen bonds between the functional groups of P(3HB), PLA, and CS, which leads to a certain restructuring of the polymer matrix and a change in the local environment of the vibrational groups. Thus, the decrease in intensity in this specified region reflects both the growing proportion of chitosan and the strengthening of interphase interaction in the multicomponent system. So, the presence of hydrogen bonds in chitosan composites was confirmed using FTIR spectra.

## 4. Conclusions

Bone tissue engineering is actively developing due to the use of biocompatible and biodegradable polymers. These include poly(3-hydroxybutyrate), the mechanical properties of which can be improved by copolymerization and the introduction of functional fillers. This study demonstrates that the formation of PHA copolymers with branched side chains, as well as the modification of P(3HB)/PLA systems with chitosan and nanohydroxyapatite, significantly affect their microstructure and properties. It is shown that the introduction of chitosan (30 wt.%) decreases the melting temperature of P(3HB) by 10.3 °C due to interfacial interaction and a reduction in crystallinity, whereas hydroxyapatite (30 wt.%) increases the hardness of the composite (from 74.0 to 79.5 Shore D), indicating effective material reinforcement.

Copolymerization of P(3HB) via the introduction of 10 mol% of 4-hydroxybutyrate units significantly enhanced the material’s plasticity, increasing elongation at break from 3.8 to 21%. Similarly, in the amorphous P(3HB)/PLA system, the addition of 30 wt.% chitosan contributed to an increase in relative elongation from 6.8 to 10.0%, which is attributed to the modification of the interfacial structure and increased segmental mobility of the polymer chains. It was established that HA is an effective reinforcing filler that improves mechanical characteristics at moderate contents and significantly influences the thermal behavior of materials depending on the crystallinity of the polymer matrix.

SEM analysis revealed a close relationship between fracture morphology and composite properties: a homogeneous structure corresponds to high strength (28.8–42.5 MPa) and elongation (3.8–6.8%), while agglomeration and porosity at 30–70% HA content cause brittle fracture, a decrease in strength to 19.7–31.3 MPa, and a reduction in thermal stability to 252–263 °C. Chitosan additionally modifies the interfacial interaction, improving structural integrity at optimal concentrations (10–20 wt.%).

The scientific novelty of this work lies in the comprehensive establishment of the relationship between fracture morphology, mechanical, and thermal properties of P(3HB)-based composites containing PLA, hydroxyapatite, and chitosan across a wide range of filler contents over a wide range of filler contents. This allows for the determination of optimal compositional regions that offer a balance of strength, plasticity, and stability.

From a practical perspective, these materials show promise for the fabrication of biodegradable filaments for 3D printing of bone tissue scaffolds, temporary implants, and osteoconductive matrices. Future research should be directed towards evaluating the biological response of cells, in vitro and in vivo degradation kinetics, as well as optimizing filler dispersion to prevent agglomeration at high concentrations. This paves the way for the creation of functionally graded biomaterials with predictable properties for regenerative medicine.

## Figures and Tables

**Table 1:** Thermal properties of extruded filaments.

Filament	T_g1_, °C	T_g2_, °C	Tcc, °C	T_m2_, °C	ΔH_m_^2^, J/g	T_deg_, °C
P(3HB)	57.9 ± 0.3	2.4 ± 0.2	57.5 ± 0.2	151.2 ± 0.3	57.6 ± 0.2	282.0 ± 0.2
PLA	64.6 ± 0.3	60.7 ± 0.3	101.6 ± 0.4	175.1 ± 0.2	38.8 ± 0.3	364.7 ± 0.2
P(3HB)/PLA (50/50)	63.88 ± 0.4	2.7 ± 0.2	96.8 ± 0.2 64.2 ± 0.4	173.8 ± 0.3 147.7 ± 0.5	24.7 ± 0.2 26.9 ± 0.4	286.3 ± 0.2 361.2 ± 0.2
P(3HB)/PLA+ 10% CS	66.8 ± 0.2	2.8 ± 0.3	96.2 ± 0.3 65.4 ± 0.2	172.7 ± 0.4 146.0 ± 0.3	27.9 ± 0.5 20.3 ± 0.2	282.4 ± 0.2 361.5 ± 0.2
P(3HB)/PLA+ 20% CS	62.4 ± 0.4	3.4 ± 0.2	58.4 ± 0.3	137.7 ± 0.2	49.4 ± 0.4	274.3 ± 0.2
P(3HB)/PLA+ 30% CS	58.9 ± 0.5	3.8 ± 0.2	86.0 ± 0.2 56.9 ± 0.3	172.5 ± 0.4 140.9 ± 0.3	21.9 ± 0.2 20.0 ± 0.5	269.1 ± 0.2 316.8 ± 0.2

Note: T_g1_ тa T_g2_—glass transition temperatures, Tcc—cold crystallization temperature, T_m2_—melting temperature, ∆Hm_2_—melting enthalpy, T_deg_—degradation temperature, P(3HB)—Poly(3-hydroxybutyrate), PLA—polylactic acid, CS—chitosan, chitosan content is indicated in weight percent (wt.%) relative to the total composite mass.

**Table 2:** Thermal properties of extruded filaments–P(3HB)/HA composites.

Filament	Shore D Hardness
P(3HB)	74.0 ± 0.5
P(3HB) + 10% HA	76.5 ± 0.5
P(3HB) + 20% HA	78.5 ± 0.5
P(3HB) + 30% HA	79.5 ± 0.5

Note: the hydroxyapatite content is indicated in weight percent (wt.%) relative to the total composite mass.

## Data Availability

The original contributions presented in this study are included in the article. Further inquiries can be directed to the corresponding authors.

## References

[1] Martina M., Hutmacher D.W. (2007). Biodegradable polymers applied in tissue engineering research: A review. Polym. Int..

[2] Bacakova L., Zarubova J., Travnickova M., Musilkova J., Pajorova J., Slepicka P., Kasalkova N.S., Svorcik V., Kolska Z., Motarjemi H. (2018). Stem cells: Their source, potency and use in regenerative therapies with focus on adipose-derived stem cells—A review. Biotechnol. Adv..

[3] Slepicka P., Kasalkova N.S., Siegel J., Kolska Z., Bacakova L., Svorcik V. (2015). Nano-structured and functionalized surfaces for cytocompatibility improvement and bactericidal action. Biotechnol. Adv..

[4] Wu S., Liu X., Yeung K.W., Liu C., Yang X. (2014). Biomimetic porous scaffolds for bone tissue engineering. Mater. Sci. Eng. R Rep..

[5] Senra M.R., Marques M.D.F.V. (2020). Synthetic polymeric materials for bone replacement. J. Compos. Sci..

[6] Sudesh K., Abe H., Doi Y. (2000). Synthesis, structure and properties of polyhydroxyalkanoates: Biological polyesters. Prog. Polym. Sci..

[7] Philip S., Keshavarz T., Roy I. (2007). Polyhydroxyalkanoates: Biodegradable polymers with a range of applications. J. Chem. Technol. Biotechnol. Int. Res. Process Environ. Clean Technol..

[8] Zinn M., Witholt B., Egli T. (2001). Occurrence, synthesis and medical application of bacterial polyhydroxyalkanoate. Adv. Drug Deliv. Rev..

[9] De Paula Cavalcante M., de Menezes L.R., da Rocha Rodrigues E.J., Tavares M.I.B. (2022). In vitro characterization of a biocompatible composite based on poly (3-hydroxybutyrate)/hydroxyapatite nanoparticles as a potential scaffold for tissue engineering. J. Mech. Behav. Biomed. Mater..

[10] Koller M., Mukherjee A. (2020). Polyhydroxyalkanoates–linking properties, applications, and end-of-life options. Chem. Biochem. Eng. Q..

[11] Wang Y., van Putten R.J., Tietema A., Parsons J.R., Gruter G.J.M. (2024). Polyester biodegradability: Importance and potential for optimisation. Green Chem..

[12] Gigante V., Cinelli P., Seggiani M., Alavarez V.A., Lazzeri A. (2020). Processing and thermomechanical properties of PHA. The Handbook of Polyhydroxyalkanoates.

[13] Müller A.J., Michell R.M. (2016). Differential scanning calorimetry of polymers. Polymer Morphology: Principles, Characterization, and Processing.

[14] Ma C., Ma Z., Yang F., Wang J., Liu C. (2019). Poly (propylene fumarate)/β-calcium phosphate composites for enhanced bone repair. Biomed. Mater..

[15] De Pace R., Molinari S., Mazzoni E., Perale G. (2025). Bone regeneration: A review of current treatment strategies. J. Clin. Med..

[16] Kaur G., Kumar V., Baino F., Mauro J.C., Pickrell G., Evans I., Bretcanu O. (2019). Mechanical properties of bioactive glasses, ceramics, glass-ceramics and composites: State-of-the-art review and future challenges. Mater. Sci. Eng. C.

[17] Song R., Murphy M., Li C., Ting K., Soo C., Zheng Z. (2018). Current development of biodegradable polymeric materials for biomedical applications. Drug Des. Dev. Ther..

[18] Jaidka S., Sharma R., Kaur S., Singh D.P., Kamaraj S.K., Thirumurugan A., Dhanabalan S.S., Hevia S.A. (2022). Scanning Electron Microscopy (SEM): Learning to Generate and Interpret the Topographical Aspects of Materials. Microscopic Techniques for the Non-Expert.

[19] Jinnai H. (2022). Electron microscopy for polymer structures. Microscopy.

[20] Pokynbroda T., Semeniuk I., Gąszczak A., Szczyrba E., Semenyuk N., Skorokhoda V., Pyshyev S. (2025). Study of Biosynthesis and Biodegradation by Microorganisms from Plastic-Contaminated Soil of Polyhydroxybutyrate Based Composites. J. Renew. Mater..

[21] Kangashaka I., Punkari T., Kattainen A., Mevada C., Keskinen J., Mäntysalo M., Sarlin E. (2025). Assessing the Performance of Biopolymers as Substrates for Flexible Printed Supercapacitors Containing Liquid Electrolytes. Adv. Energy Sustain. Res..

[22] (2022). Standard Test Method for Tensile Properties of Plastics.

[23] (2015). Standard Test Method for Rubber Property—Durometer Hardness.

[24] Kohan M., Lancoš S., Schnitzer M., Živčák J., Hudák R. (2022). Analysis of PLA/PHB biopolymer material with admixture of hydroxyapatite and tricalcium phosphate for clinical use. Polymers.

[25] Qu X.H., Wu Q., Chen G.Q. (2006). In vitro study on hemocompatibility and cytocompatibility of poly(3-hydroxybutyrate-co-3-hydroxyhexanoate). J. Biomater. Sci. Polym. Ed..

[26] Gonciarz W., Balcerczak E., Brzeziński M., Jeleń A., Pietrzyk-Brzezińska A.J., Narayanan V.H.B., Chmiela M. (2025). Chitosan-based formulations for therapeutic applications. A recent overview. J. Biomed. Sci..

